# Does Physical Fitness Affect Academic Achievement among Japanese Adolescents? A Hybrid Approach for Decomposing Within-Person and Between-Persons Effects

**DOI:** 10.3390/ijerph15091901

**Published:** 2018-09-01

**Authors:** Akira Kyan, Minoru Takakura, Masaya Miyagi

**Affiliations:** 1 Graduate School of Health Sciences, University of the Ryukyus, 207 Uehara, Nishihara, Okinawa 903-0215, Japan; kyankyan67@gmail.com; 2 Faculty of Medicine, University of the Ryukyus, 207 Uehara, Nishihara, Okinawa 903-0215, Japan; 3 Faculty of Education, University of the Ryukyus, 1 Senbaru, Nishihara, Okinawa 903-0213, Japan; masaya@edu.u-ryukyu.ac.jp

**Keywords:** academic performance, sex characteristics, socio-economic status, Japan, longitudinal study

## Abstract

Positive association between physical fitness and academic achievement in adolescents has been suggested yet the causal effect of physical fitness on academic achievement remains unclear. This study examined if longitudinal changes in physical fitness were associated with changes in academic achievement among junior high school students. Analyses were based on a two-year with three time-point data of 567 students (aged 12–13 years old at the baseline-point; 303 boys) who entered in five Japanese junior high schools in 2015. Academic achievement was evaluated using the student’s overall grade point average. Comprehensive physical fitness score was summed up from eight fitness tests: 50-m sprint, standing broad jump, repeated side-steps, sit and reach, sit-ups, hand-grip strength, handball throw, and 20-m shuttle run or endurance run. The hybrid regression model was applied to examine the impact of change in physical fitness on change in academic achievement using multiple imputation to account for non-response at follow-up. The changes in fitness score within-person and the differences in average of fitness score of three-time points between-person were associated with change in overall grade point average for boys. No significant association between fitness score and overall grade point average was observed in girls. Opportunities for increased physical fitness may be important to support academic achievement, particularly in junior high school boys.

## 1. Introduction

In the last few decades, a growing body of research has built an argument that physical fitness (PF) among youth might affect their cognitive development and academic achievement (AA) [1]. With regard to the possibility of the effects of PF on cognition and AA in children, Tomporowski et al. [2] have proposed a hypothetical conceptual model. In the model, they identified psychosocial factors, health factors, and PF as direct precursors to children’s mental functioning (e.g., academic achievement), and suggested that socioeconomic status (SES) level and gender may moderate as well [2]. Among them, PF has historically been regarded as a potential mediator of the effects of exercise training on cognitive functions/structure [2]. To date, some studies found that children with higher aerobic fitness exhibited larger hippocampal volumes as well as superior performance on a relational memory task compared to those with lower fitness [3]. Other studies observed that the basal ganglia, which support executive function, are larger in higher fit children relative to lower fit ones. Also, higher fit children exhibited better behavioural performance during a task requiring the modulation of executive function [1]. Since these brain functions are considered a fundamental factor influencing AA [1,3,4,5], it has been suggested that promoting PF can lead to better AA [1,6].

Although many cross-sectional studies supported the positive correlation between PF and AA [1,6], the causal effect of PF on AA remains unclear due to few longitudinal studies [1,6]. A recent review by Santana et al. [6] revealed that of the 45 observational studies investigating the PF-AA relationship published from 1990 to 2015, only three employed longitudinal designs which examined the association between comprehensive PF and AA. Of these, Bezold et al. [7] examined the longitudinal relationship of PF to AA using data of 83,111 New York City middle-school students. They reported that a substantial increase in PF from previous years resulted in a greater improvement in AA than was seen among those with no to minimal change of PF in both boys and girls. On the other hand, London and Castrechin [8] demonstrated that an academic gap between consistently fit and consistently unfit students did not change over time. Therefore, more research is needed to investigate how changes in PF may be related to subsequent changes in academic outcomes.

One of the advanced analytical techniques to assess change that is systematically related to the passage of time is using hybrid models [9,10,11]. The model combines the advantage of random-effects and fixed-effects panel analysis. The great advantage of hybrid models is that these estimate two coefficients for each variable: a person-level mean which captures between-person effects, and a measure of the deviation of each observation from the person-specific mean which captures the within-person effects. The approach can provide unbiased estimates of time constant variables even in the presence of unobserved heterogeneity, and provide estimates for time varied variables [9,10,11]. This model recently has received a lot of attention as one of the novel panel data analysis in the research field of sociology or economics [12]. By applying hybrid models in this study, it will be able to achieve a more reliable and unbiased estimation of how changes in PF affect AA by controlling for both time varied and time constant variables simultaneously. To the best of our knowledge, however, no previous study has examined the causal effects of PF on AA using the hybrid models.

Examining the relationship between PF and AA, SES and individual psychological characteristics such as motivation are considered as the time constant variables observed between persons [1,2,6]. SES is generally known to have substantial impacts on academic performance [13] and have been suggested to moderate the association between PF and AA [1,2]. Nevertheless, it has been pointed out that few studies have controlled for SES [1,6], and indeed some studies found no or weak association between PF and AA after adjusting for SES [14,15,16]. Therefore, it is necessary to take SES into consideration for detecting the effects of PF on AA. On the other hand, a key individual psychological characteristic related to the association between PF and AA is achievement motivation. Achievement motivation is a theoretical model intended “to explain how the motive to achieve and the motive to avoid failure influence behaviour [17]” and is one of the psychological characteristics reflecting the human desire to do things well and overcome obstacles [18]. According to Mori and Horino [19], achievement motivation involves the pursuit of goals evaluated by one’s own standards of achievement regardless of social and cultural values. This consists of two aspects: self-fulfilment achievement motivation (SFAM) and competitive achievement motivation (CAM). SFAM is a disposition in which an individual desire to attain their own goals, whereas CAM is a disposition in which an individual desire to seek social prestige by defeating others. Achievement motivation plays an important role in achieving high performance at school [20], engaging in physical activity continuously on a daily basis, [21] and striving in field tests of PF [22,23]. Indeed, a recent cross-sectional study has indicated that the relationship between PF and AA might be affected by achievement motivation [24]. However, few longitudinal studies have examined the effects of PF on AA, accounting for the potential confounders simultaneously.

The changeable factors with aging are needed to take into consideration, particularly in adolescents because adolescence is in the midst of puberty and is the period of rapid physical, cognitive, and social maturation [25]. The underlying biological processes resulting in physical changes during puberty have intellectual, emotional, social and behavioural implications [26]. The time varied covariate required to be accounted for in examining the PF-AA relationship is weight status (e.g., body mass index (BMI)). This is because, as with PF, BMI changes with physical maturation or age during adolescence. BMI correlates to PF and has been suggested to be negatively associated with AA [27,28,29]. Changes in BMI thus should be considered as a time varied confounder on the relationship between PF and AA. Furthermore, another crucial factor for examining the determinants of AA is learning duration. It is generally known that setting aside time to study out of school leads to high AA [24,30,31]. Nevertheless, previous prospective studies examining PF-AA relationship have not accounted for learning duration. By filling up the lack of essential confounding factors, it might even negate the relationship between PF and AA.

To address the concerns mentioned above, therefore, the aim of this study is to examine the causal impact of PF on AA of junior high school students in Japan, while taking into account essential time constant covariates including SES and individual psychological characteristics and time varied covariates including BMI and learning duration. Moreover, we also examined the sex differences on the relationship because the relations might be moderated by gender [2]. Although the different association between boys and girls has been shown mixed results [1,6], two recent cross-sectional studies in Japanese adolescents have reported the positive relations only in boys [24,30]. We thus hypothesized that PF would positively affect AA only in boys.

## 2. Materials and Methods

### 2.1. Procedure and Participants

**Figure 1:**
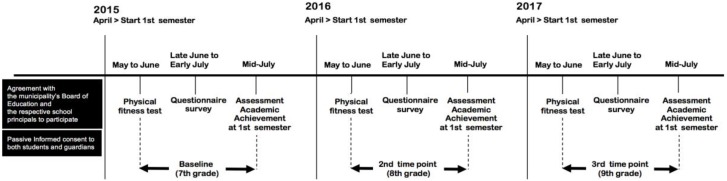
Procedure of this study.

Figure 1 shows the procedure of this study. A two-year, with three-time points longitudinal study was conducted in five public junior high schools in two suburban municipalities in Okinawa Prefecture, Japan from April 2015 to July 2017. Prior to the conduct of the study, we obtained an agreement with the municipality’s Board of Education and the respective school principals to participate in the study. A total of six schools in the two municipalities were invited to participate in the study. Five schools agreed to participate. Passive informed consent was also obtained from the parents/guardians at the first year of this study. The students were requested to take home the informed consent form which provided the information regarding the ethical considerations of the study. The study was conducted in accordance with the Declaration of Helsinki, and the protocol was approved by the Ethics Committee of the University of the Ryukyus (authorization number: 341). Participation of the students was entirely voluntary, and the confidentiality of the participants information was ensured. Students were also free to decline to participate in the study. The parents/guardians were given the phone number and e-mail address of the principal investigator (corresponding author of this article) and had the opportunity to withdraw their children from participation to the study by declaration. All assenting students who had their parents’ consent were requested to complete and return the questionnaire sealed in an unmarked envelope to assure the confidentiality of their responses. The Ethics Committee of the University of the Ryukyus approved the study protocol of this study and formally waived the requirement for written consent (authorization number: 341).

Of those 613 seventh grade students (aged 12–13 years old) enumerated at the beginning of this research, 605 students (98.7% of the original sample; 326 boys) were followed up. Classroom teachers distributed the self-administered questionnaires between June and July for every year as prescribed by the researchers. The questionnaires consisted of questions about socio-demographic attributes, psychosocial school environment, lifestyle and health status. Data about PF and AA were obtained from school records at the end of the first semester in mid-July of each year. Finally, we collected the data of 567 students (92.4% of the original sample; 303 boys) through three-time points. Students who did not submit the questionnaires and had incomplete PF and AA data were excluded.

### 2.2. Measures

#### 2.2.1. Academic Achievement

Academic achievement was evaluated using the student’s overall grade point average (GPA) for Japanese, Mathematics, Science, Social Studies, and English subjects. These subjects have to be learned by junior high school students and are commonly used for the entrance examination in high school. Each student was rated on a five-point scale for each subject by school teachers. School teachers assessed the students based on the evaluation standards set out in the Japanese government’s curriculum guidelines. This method of assessment is being implemented in almost all junior high schools in Japan and is generally used as an evaluation indicator in entrance examinations for high school. However, as the evaluation standards are commonly adjusted to the educational level of students of each school, the mean and variance of grades differ. These differences were observed in the current study (data not shown). Therefore, we standardized the GPA to z-score by each school. The z-score measures the number of standard deviations above or below the mean score and gives us a way to normalize the data consistently across years [8].

#### 2.2.2. Physical Fitness

Physical fitness was assessed using the New Physical Fitness Test. It is a Japanese national survey implemented by Ministry of Education, Culture, Sports, Science, and Technology of Japan (MEXT). The fitness test was composed of the following: 50-m sprint, standing broad jump, repeated side-steps, sit and reach, sit-ups, hand-grip strength, handball throw, and 20-m shuttle run or endurance run (1500 m for boys; 1000 m for girls) [32]. Elective choice between 20-m shuttle run and endurance run was allowed in each school [32]. Each measured value was converted into a score ranging from 1 to 10 for each sex based on performance-to-score conversion tables standardized by the Japanese nationwide survey [32]. Then, these were summarized as a total score (range of 8 to 80). All measurements were conducted by the Physical Education teachers at each school in accordance with the test manual, which had been released by MEXT [32].

### 2.3. Covariates

#### 2.3.1. Body Mass Index

Body mass index was calculated as weight (kg)/height (m)^2^, data which were obtained from school records. Body measurements were taken by school nurse-teachers as part of the standard procedure carried out every April to June in Japan for school records. During analysis, BMI was treated as a continuous variable.

#### 2.3.2. Achievement Motivation

Achievement motivation was assessed using an achievement motive scale developed by Mori and Horino [19]. The scale was composed of the aforementioned two factors: SFAM and CAM. The validity and reliability of the achievement motive scale has been verified among Japanese children [19]. This scale comprised of 10 items for each aspect. The response format was 1 = “Strongly agree”, 2 = “Agree”, 3 = “Disagree”, and 4 = “Strongly disagree”. The total score for each aspect was calculated and treated as a continuous variable. In the current sample, the Cronbach ’s α coefficients for SFAM and CAM were 0.87 and 0.86, respectively. The achievement motive variable collected at the first year was used in the analysis.

#### 2.3.3. Learning Duration

Learning duration was assessed by asking the time spent on learning after school, including cram school or private teacher, on weekdays. Respondents selected one of the following options: “1 = None”, “2 = Less than 30 min”, “3 = 30–60 min”, “4 = 1–2 h”, “5 = 2–3 h”, and “6 = 3–4 h”. The option number was used in the analysis. The learning duration data was self-reported at three-time points.

#### 2.3.4. Socio-Economic Status

The present study used family structure and parental education level as proxies of SES. Family structure was assessed using information regarding all people living at home, coded as “living with both parents” or “other”. This was collected at the start of this research and was dummy coded with those of living with both parents as the reference during analysis. Parental education level was assessed by asking the highest education level attained by the student’s mother or father. Categories were “junior high school or high school”, “specialized training college or junior college”, and “university or higher”, based on the international standard classification of education [33]. The parental education level data was collected once at the first year and dummy coded with university as the reference when using it in the analysis.

### 2.4. Analysis

Descriptive analysis was applied to examine study subject’s characteristics at three-time points, and changes for PF, BMI, learning duration, and GPA, and SES variables and motivation at first-time points. For the hybrid regression model, we resolved PF, BMI, and learning duration into within-person and between-persons components. To examine the association between bivariate PF and AA, model 1 includes only PF at both components. Subsequently, the confounding factors were added into model 1 for model 2. The between-person effects generated conclusions on whether AA is associated with three time-point averages of PF. The within-person effects generated conclusions about the impacts of three time-point changes of PF within-person by comparing the change of GPA in two years. All analyses were conducted separately for boys and girls. To complement the missing values, we adopted multivariable multiple imputation (MI). We generated 25 imputed datasets and combined estimates across these using Rubin’s rules [34,35]. The variables in the imputation model were school, sex, grade, height, weight, BMI, PF score, GPA (*z*-score), learning duration, achievement motive, presence of both parents, and parental education. Data analysis was performed using IBM SPSS statistics 25.0 (IBM Co., Tokyo, Japan). The level of statistical significance was set at *p* < 0.05.

## 3. Results

### 3.1. Characteristics of Study Participants

The characteristics of study participants over two years are shown in Table 1. The change of the average weight and height with the advance of students’ grade was similar to this population [36]. The total physical fitness score increased every year in both sexes. The percentages of students spending one or more hours to learn outside of school were lowest at the second time point in both sexes. In terms of the family structure, approximately 75% lived with both parents among both boys and girls. The proportion that parental education level in the MI model was university or higher was 35.6% in boys and 27.8% in girls.

### 3.2. Finding from the Hybrid model analysis

Table 2 presents the results of the hybrid model analysis using imputed data separately for boys and girls. The within-person coefficients showed that the change of total fitness scores through two-year time periods is associated with a change in GPA for boys (beta = 0.007, standard error = 0.002, 95 % confidential interval = 0.002–0.011). For girls, the changes of total fitness score showed no significant impact on GPA according to within-person effect. The between-person coefficients showed that total fitness scores on the average of two years is positively associated with GPA only for boys (beta = 0.026, standard error = 0.006, 95% confidential interval = 0.014–0.037). No significant relation in total fitness score and GPA was observed in girls but at least PF did not negatively affect AA. These associations were observed after adjustment for BMI, learning duration, achievement motive, absence of parents, and parental education. Between-person differences in learning duration showed significantly positive association with AA in both sexes (boys: beta = 0.149, standard error = 0.052, 95% confidential interval = 0.047–0.251, girls: beta = 0.191, standard error = 0.051, 95% confidential interval = 0.091–0.292) but the within-person change is positive only in girls (beta = 0.079, standard error = 0.025, 95% confidential interval = 0.031–0.128). Having a single parent negatively affected AA in both boys (beta = −0.386, standard error = 0.109, 95% confidential interval = −0.600–−0.171) and girls (beta = −0.236, standard error = 0.110, 95% confidential interval = −0.452–−0.020). Parental education level with less than high school negatively affected AA for boys (beta = −0.227, standard error = 0.092, 95% confidential interval = −0.410–−0.043) and girls (beta = −0.343, standard error = 0.104, 95% confidential interval = −0.550–−0.137).

## 4. Discussion

The main finding of this study was that a positive effect of PF on AA was observed only among boys. Importantly, aside from consistently having high-fitness status, favourable change of PF within the individual through the two-year study period certainly contributed to AA of boys, even after adjusting several potential confounding factors. Therefore, opportunities for increased PF may be important to support AA among junior high school boys, regardless of academic gap. These findings support previous studies which suggest the benefit to AA by enhancing PF [7,37].

One biological mechanism that can explain how PF may improve AA is the induction of physiological and psychological changes through various types of physical exercise. These changes have an important role in AA. The view that aerobic exercise and motor-related activities can induce physiological changes in the brain has been verified. To date, it has been suggested that engagement in aerobic exercise increases cerebral blood flow and arousal levels [38], induces tranquilizing effect of neurotransmitters (noradrenaline, adrenaline, and serotonin) [39], and expedites development brain-derived neurotrophic factor related growth and plasticity of neurons [40], promotes angiogenesis and neurogenesis in the hippocampus which is the part of the brain responsible for memory [3], and has the potential to induce vascularization and neural growth and to alter synaptic transmission in the prefrontal cortices in those regions of the brain tied to executive function [1,41]. Likewise, Aadland et al. [42] suggested a possibility that such neurogenesis in the hippocampus and physiological changes could be induced by gains obtained in motor skills performance through performing gross and complex motor tasks. Although this structural modification hypothesis by motor task is unevidenced in children [1], there are evidences which show that coordination training is related to hippocampal volume increases [43] and grey matter increases in prefrontal region in adult [44]. The hippocampus refers to the hub of a network involving prefrontal and parietal regions that supports effective learning strategies [45], and executive function ruled by prefrontal cortices. This plays an important role in numerous educational settings and domains such as reasoning, mathematical problem solving, and language comprehension [46]. Although we cannot identify which fitness component could lead to substantial AA, we infer that enforcement of PF through various types of physical exercise leads to improvement of cognitive function/structure, thereby resulting to a positive impact on AA.

Also, some psychological factors, such as self-esteem [47,48,49], depressed mood [47], and self-efficacy [50], have been reported to mediate the relationship between PF and AA. Particularly, success in exercise is associated with various psychological and cognitive positive influences [51]. For example, studies focusing on the effects of participation in organized team sports activities indicated that it increases self-esteem [52] and decreases social anxiety [53]. Additionally, it has been suggested that cognitive demands inherent in goal-directed physical activities (e.g., group games) also develop cognitive skills that can also be used for executive function tasks [54].

In Japanese junior high school students, extracurricular sports activity (ESA) is an opportunity to enforce their PF. Japanese ESA is internationally unique in that it belongs to schools and commonly administered by school teachers, whereas youth sports are centred on community clubs outside schools in other countries [55]. The course of study by MEXT stipulates that ESA is part of school educational activity and school teachers encourage students’ motivation for learning, cultivation of a sense of responsibility, and feelings of solidarity [56]. In other words, students who participate in ESA are educated to develop their personalities. According to an annual national survey in 2017 by the National Institute for Educational Policy Research, 67.3% of third grade junior high school students belonged to ESA at their schools and the rate of engaging in ESA for 1 h or more per day reached 83.7% [57]. Another set of national data showed that ESA is held more than five days per week [58]. Given the certain differences in fitness status between participants and non-participants reported in the national survey [57], it is inferred that variance of PF change in the present study reflected participation/non-participation in ESA. Thus, the association of PF and AA of boys shown in this study might be explained by participation in ESA. Consequently, in addition to aforementioned physiological and psychological effects of physical exercise, educational encouragement by teacher during ESA may lead to academic development in boys. Meanwhile, this explanation is nothing more than a possibility. Causal effects of Japanese ESA on AA have not been investigated, so far. Further study in this regard is needed.

In contrast to the positive effect of PF on AA for boys, no significant association between PF and AA was observed among girls. The results supported our hypothesis that the causal impact of PF on AA would be seen only in boys. Morita and colleagues [24,30] have reported the positive associations of PF with AA among boys only, whereas BMI associated with AA negatively in girls. Our results expanded the possibility that effects of PF to AA in Japanese adolescents’ population are seen only among boys. On the other hand, the possibility that girls’ BMI is an obstructive factor of AA was dismissed. Although the possible effect of BMI on AA remains controversial [27,28,31,59,60,61], at least, our results suggested that it is not a direct causal relationship.

As a reason for such sex difference in Japanese adolescents, a possibility has been suggested: predicting AA model by psychological (i.e., achievement motive), behavioural (i.e., learning duration, screen time, and exercise habit), and physical (i.e., cardiorespiratory fitness and BMI) factor differs between boys and girls [24]. According to the model, two pathways of achievement motivation to GPA are described in boys: (1) SFAM → screen time/learning duration → GPA, and (2) CAM → exercise habit → CRF → GPA; and in females, the two pathways are: (1) SFAM → screen time/learning habit → GPA, and (2) exercise habit → BMI^2^ → GPA [24]. Given the finding, achievement motivation showing no significant effect on AA in the present study might relate to AA indirectly via behavioural factors. Further research is needed to better understand the role which achievement motivation plays in mediation factors, including behaviour, underlying between PF and AA.

The sex difference was consistent with some previous findings in other countries [6,24]. Assuming that the sex differences are general, a possible explanation for the differences between the sexes is that exercise-induced improvement of cognitive functions is sexually dimorphic in children. Drollette et al. [62] found that higher aerobic fitness levels were associated with better working memory performance only among boys. They attributed the observed sexual dimorphism with the inhibitory effect of increased oestrogen levels among girls of puberty age on the expression of exercise-induced brain-derived neurotrophic factors in the hippocampus [62]. Moreover, the results of one study suggested that there are differences in neural networks related to motor functions and language between boys and girls [63]. Haapala et al. [16] likewise reported that biological maturation between sexes might explain the relationship between motor performance and AA. Collectively, although highly speculative, the relationship between PF and AA may exhibit sex-specific differences due to sex dimorphism in exercise-induced improvement of cognitive structure/functions attributed to biological maturation differences between girls and boys. Previous literature, however, have mixed results on sex differences in the association between PF and AA [1,6], and its underlying relationship with PF and AA remain unclear [1]. Thus, future studies are needed to confirm this and elucidate the possible explanatory mechanism.

Looking at the regression coefficients of each independent variable in our results, SES variables have the largest impact on AA for both sexes. A large body of research has found adolescents from lower SES have an increased risk for poor educational outcomes [13]. In this context, Lee [64] described two explanatory mechanisms underlying this SES effect: (1) lower SES students are less likely to recognize the implications of their choice and (2) low-SES families are less likely to seek out all the information they need, and to handle the complexity of the information to make appropriate educational choices. While educational disadvantage by SES can be also found in our results as with the previous findings, we demonstrated that improving health behaviour by enhancing PF through sports and physical exercise could lead to educational supports regardless level of SES. In Japan, there are relatively few empirical evidences on educational performance as compared with other countries, because information disclosure on education has been extremely limited [65]. Our findings can be a valuable evidence for the education sector.

### Limitations

These findings cannot elucidate the mechanisms that link PF and AA. It also should be noted that our study sample was limited in a suburban area in Okinawa Prefecture, which may affect its generalizability. Future studies are needed to investigate the relations between PF and AA considering the potential intermediation factors such as cognitive function using a large sample.

## 5. Conclusions

By using the hybrid model analysis, we detected a possibility that an increase in PF leads to good AA among junior high school boys, regardless of between-person differences of PF level. Moreover, it is suggested that the effect of PF on AA is effective without negative influences by SES. Although the lack of relationship in PF and AA was observed in girls, PF is unlikely to negatively influence AA. Collectively, opportunities for promoting PF may support AA among junior high school students. The research pursuing sex differences is insufficient in this research field [1,6]. More research is needed to examine the moderation effect by sex and elucidate the possible explanatory mechanism.

## Figures and Tables

**Table 1:** Participants’ characteristics at each of the study periods.

Variable		Pre-Imputation			Post Imputation (Imputed 25 Datasets)		
		7th Grade	8th Grade	9th Grade	7th Grade	8th Grade	9th Grade
Boys		(n = 200)	(n = 190)	(n = 192)	(n = 303)	(n = 303)	(n = 303)
Height [Mean (S.E.)]		152.12 (0.56)	159.41 (0.53)	164.57 (0.46)	151.72 (0.51)	159.25 (0.44)	164.26 (0.39)
Weight [Mean (S.E.)]		44.26 (0.74)	49.27 (0.79)	54.54 (0.84)	43.64 (0.55)	48.84 (0.58)	53.37 (0.59)
BMI [Mean (S.E.)]		18.95 (0.23)	19.26 (0.24)	20.07 (0.27)	18.80 (0.18)	19.15 (0.18)	19.72 (0.19)
GPA (Z-score) [Mean (S.E.)]		−0.13 (0.07)	−0.01 (0.07)	−0.08 (0.07)	-0.20 (0.06)	−0.14 (0.05)	−0.15 (0.06)
Total fitness score [Mean (S.E.)]		35.62 (0.59)	44.77 (0.72)	48.80 (0.74)	35.72 (0.51)	44.02 (0.56)	48.18 (0.59)
SFAM [Mean (S.E.)]		3.19 (0.04)			3.15 (0.04)		
CAM [Mean (S.E.)]		2.98 (0.05)			2.97 (0.04)		
Learning duration [n (%)]							
	none	8 (4.0)	24 (12.6)	9 (4.7)	17 (5.5)	36 (12.0)	21 (6.9)
	Less than 30 min	27 (13.5)	34 (17.9)	35 (18.2)	36 (11.9)	52 (17.1)	51 (16.8)
	30 min to 1 h	73 (36.5)	71 (37.4)	45 (23.4)	107 (35.3)	102 (33.6)	71 (23.5)
	1 to 2 h	68 (34.0)	24 (12.6)	44 (22.9)	97 (31.9)	53 (17.3)	71 (23.6)
	2 to 3 h	13 (6.5)	25 (13.2)	37 (19.3)	26 (8.5)	39 (12.7)	57 (18.7)
	More than 3 h	11 (5.5)	12 (6.3)	22 (11.5)	21 (6.9)	22 (7.2)	32 (10.6)
Family structure [n (%)]							
	Both parents	157 (78.5)			227 (74.9)		
	Other	43 (21.5)			76 (25.1)		
Parental education level [n (%)]							
	JHS/HS	88 (44.0)			136 (44.8)		
	Spec/college	40 (20.0)			59 (19.6)		
	University	72 (36.0)			108 (35.6)		
Girls		(n = 182)	(n = 172)	(n = 170)	(n = 264)	(n = 264)	(n = 264)
Height [Mean (S.E.)]		150.72 (0.43)	153.37 (0.39)	154.71 (0.40)	150.95 (0.37)	153.58 (0.33)	154.7 (0.36)
Weight [Mean (S.E.)]		43.21 (0.58)	46.26 (0.57)	48.71 (0.55)	43.61 (0.49)	46.54 (0.46)	48.29 (0.43)
BMI [Mean (S.E.)]		18.93 (0.20)	19.62 (0.20)	20.31 (0.19)	19.06 (0.17)	19.69 (0.16)	20.15 (0.16)
GPA (Z-score) [Mean (S.E.)]		0.43 (0.06)	0.45 (0.07)	0.47 (0.06)	0.3 (0.06)	0.26 (0.06)	0.28 (0.06)
Total fitness score [Mean (S.E.)]		46.14 (0.80)	51.73 (0.82)	52.79 (0.84)	46.42 (0.64)	51.68 (0.65)	53.12 (0.65)
SFAM [Mean (S.E.)]		3.19 (0.04)			3.15 (0.03)		
CAM [Mean (S.E.)]		2.74 (0.05)			2.71 (0.04)		
Learning duration [n (%)]							
	none	6 (3.3)	7 (4.1)	9 (5.3)	10 (3.9)	18 (7.0)	22 (8.2)
	Less than 30 min	8 (4.4)	27 (15.7)	17 (10.0)	20 (7.5)	46 (17.3)	29 (11.2)
	30 min to 1 h	43 (23.6)	66 (38.4)	42 (24.7)	60 (22.8)	90 (34.1)	63 (24.0)
	1 to 2 h	73 (40.1)	48 (27.9)	38 (22.4)	99 (37.5)	72 (27.2)	60 (22.8)
	2 to 3 h	44 (24.2)	12 (7.0)	38 (22.4)	59 (22.2)	22 (8.2)	53 (20.3)
	More than 3 h	8 (4.4)	12 (7.0)	26 (15.3)	16 (6.1)	17 (6.3)	36 (13.6)
Family structure [n (%)]							
	Both parents	148 (81.3)			198 (75)		
	Other	34 (18.7)			66 (25)		
Parental education level [n (%)]							
	JHS/HS	77 (42.3)			122 (46.3)		
	Spec/college	49 (26.9)			68 (25.9)		
	University	56 (30.8)			73 (27.8)		

**Table 2:** Hybrid model predicting the academic achievement using imputed data.

			Model 1				Model 2			
			Coef.	S.E.	*p*	95% CI	Coef.	S.E.	*p*	95% CI
Boys										
Within effects										
	Total Fitness Score		0.007	0.002	0.002	(0.002; 0.011)	0.007	0.003	0.031	(0.001; 0.014)
	BMI						−0.019	0.018	0.269	(−0.054; 0.015)
	Learning duration						0.036	0.020	0.070	(−0.003; 0.074)
Between effects										
	Total Fitness Score		0.026	0.006	<0.001	(0.014; 0.037)	0.024	0.006	<0.001	(0.013; 0.034)
	BMI						−0.019	0.017	0.262	(−0.053; 0.014)
	Learning duration						0.149	0.052	0.004	(0.047; 0.251)
	SFAM						0.116	0.093	0.215	(−0.069; 0.301)
	CAM						−0.098	0.094	0.302	(−0.286; 0.090)
	Family structure *						−0.386	0.109	<0.001	(−0.600; −0.171)
	PE **:	JHS/HS					−0.227	0.092	0.016	(−0.410; −0.043)
		Spec/college					−0.106	0.096	0.270	(−0.297; 0.084)
Girls										
Within effects										
	Total Fitness Score		0.006	0.004	0.113	(−0.001; 0.014)	0.006	0.006	0.367	(−0.007; 0.018)
	BMI						0.009	0.021	0.671	(−0.033; 0.051)
	Learning duration						0.079	0.025	0.001	(0.031; 0.128)
Between effects										
	Total Fitness Score		0.009	0.005	0.109	(−0.002; 0.019)	0.007	0.006	0.234	(−0.004; 0.017)
	BMI						0.001	0.019	0.944	(−0.036; 0.038)
	Learning duration						0.191	0.051	0.000	(0.091; 0.292)
	SFAM						0.106	0.111	0.341	(−0.113; 0.325)
	CAM						−0.044	0.090	0.625	(−0.221; 0.133)
	Family structure *						−0.236	0.110	0.032	(−0.452; −0.020)
	PE **:	JHS/HS					−0.343	0.104	0.001	(−0.550; −0.137)
		Spec/college					−0.223	0.116	0.057	(−0.453; 0.006)

Coef. = Coefficients, BMI = Body mass index, SFAM = Self-fulfilment achievement motive, CAM = Competitive achievement motive, PE = parental education level, JHS/HS = Junior high school or high school, Spec/college = Specialized training college or junior college. * Family structure was dummy coded with those of living with both parents as the reference. ** Parental education was dummy coded with university as the reference. *Note*: Information criteria are not available, for which a multiple imputation analysis was performed.
